# Supplementary material for: Optimal minimal residual disease threshold in pediatric acute myeloid leukemia: A retrospective cohort study based on the TARGET database
Source: PLoS Med. 2026 May 8;23(5):e1005088. doi: 10.1371/journal.pmed.1005088 (PMC13155632; doi:10.1371/journal.pmed.1005088)
Supplement: S1 Code — (ZIP) [file pmed.1005088.s002.zip › S2 code/PROJ8_3_tbl1/PROJ8_3_tbl1_b.pdf]

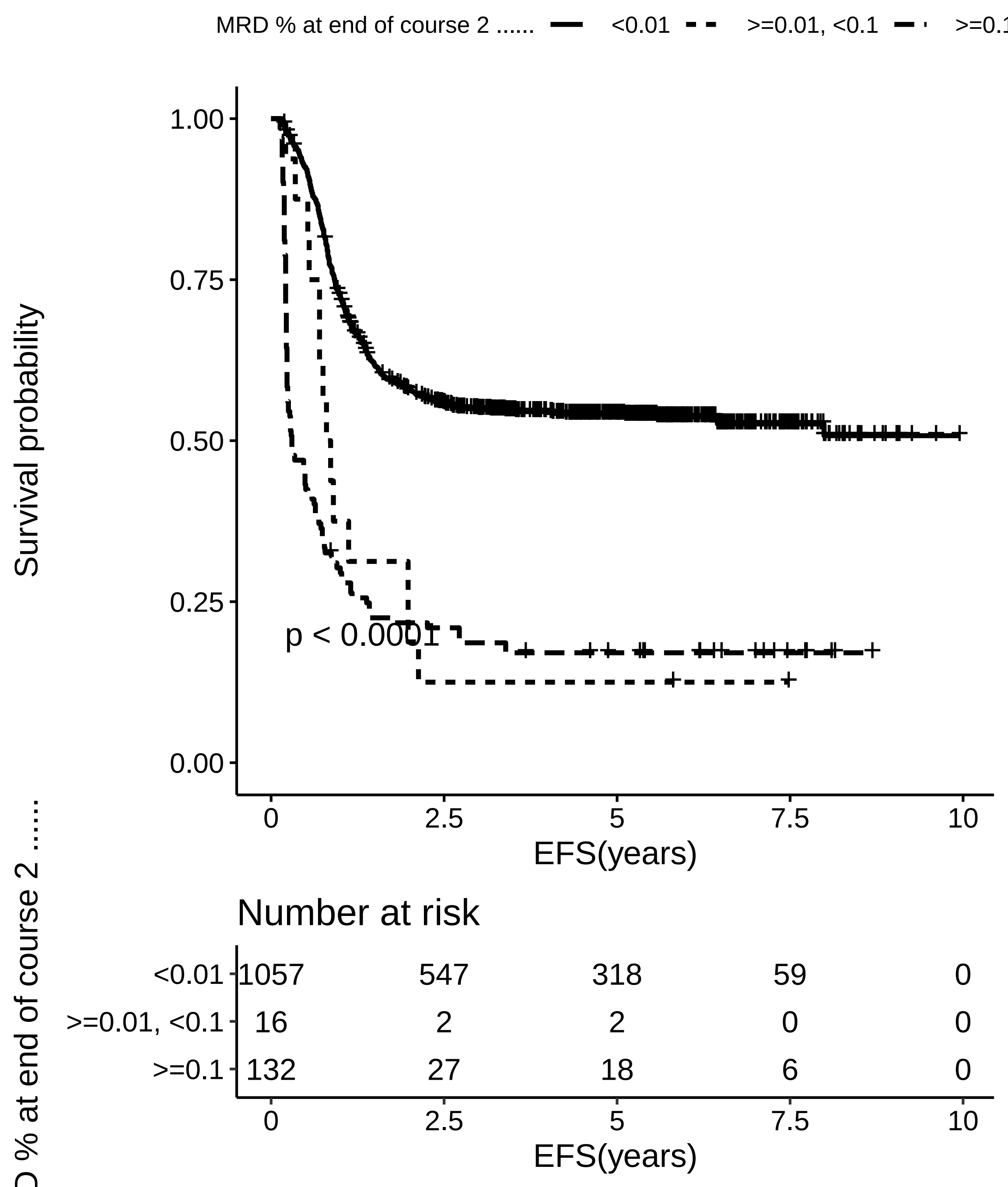
MRD % at end of course 2 ......
<0.01
>=0.01, <0.1
Survival probability
1.00
0.75
0.50
0.25
0.00
p < 0.0001
0
2.5
5
7.5
10
EFS(years)
Number at risk
<0.01 1057 547 318 59 0
>=0.01, <0.1 16 2 2 0 0
>=0.1 132 27 18 6 0
0
2.5
5
7.5
10
EFS(years)
